# Somatostatin and Astroglial Involvement in the Human Limbic System in Alzheimer’s Disease

**DOI:** 10.3390/ijms22168434

**Published:** 2021-08-05

**Authors:** Melania Gonzalez-Rodriguez, Veronica Astillero-Lopez, Patricia Villanueva-Anguita, M. Eugenia Paya-Rodriguez, Alicia Flores-Cuadrado, Sandra Villar-Conde, Isabel Ubeda-Banon, Alino Martinez-Marcos, Daniel Saiz-Sanchez

**Affiliations:** Neuroplasticity & Neurodegeneration Laboratory, Ciudad Real Medical School, CRIB, University of Castilla-La Mancha, 13071 Ciudad Real, Spain; melania.gonzalez@uclm.es (M.G.-R.); veronica.astillero@uclm.es (V.A.-L.); patricia.villanueva@uclm.es (P.V.-A.); marupayapaka@gmail.com (M.E.P.-R.); alicia.flores@uclm.es (A.F.-C.); sandra.villar@uclm.es (S.V.-C.); Isabel.Ubeda@uclm.es (I.U.-B.); Alino.Martinez@uclm.es (A.M.-M.)

**Keywords:** Alzheimer’s disease, somatostatin, hippocampus, olfactory bulb, astroglia, stereology

## Abstract

Alzheimer’s disease (AD) is the most prevalent neurodegenerative disease in the elderly. Progressive accumulation of insoluble isoforms of amyloid-β peptide (Aβ) and tau protein are the major neuropathologic hallmarks, and the loss of cholinergic pathways underlies cognitive deficits in patients. Recently, glial involvement has gained interest regarding its effect on preservation and impairment of brain integrity. The limbic system, including temporal lobe regions and the olfactory bulb, is particularly affected in the early stages. In the early 1980s, the reduced expression of the somatostatin neuropeptide was described in AD. However, over the last three decades, research on somatostatin in Alzheimer’s disease has been scarce in humans. Therefore, the aim of this study was to stereologically quantify the expression of somatostatin in the human hippocampus and olfactory bulb and analyze its spatial distribution with respect to that of Aβ and au neuropathologic proteins and astroglia. The results indicate that somatostatin-expressing cells are reduced by 50% in the hippocampus but are preserved in the olfactory bulb. Interestingly, the coexpression of somatostatin with the Aβ peptide is very common but not with the tau protein. Finally, the coexpression of somatostatin with astrocytes is rare, although their spatial distribution is very similar. Altogether, we can conclude that somatostatin expression is highly reduced in the human hippocampus, but not the olfactory bulb, and may play a role in Alzheimer’s disease pathogenesis.

## 1. Introduction

Alzheimer’s disease (AD) is the most prevalent disease in the elderly and is associated with cognitive deficiencies, including memory and olfactory impairments [1,2]. Memory loss has been related to cholinergic neurodegeneration of the temporal lobe, especially focused on the entorhinal cortex and hippocampus (Hip) [3]. The extracellular accumulation of insoluble isoforms of amyloid-β (Aβ) is the main neuropathological feature in AD, together with the aggregation of hyperphosphorylated tau protein that triggers the formation of neurofibrillary tangles, inducing intracellular toxicity and cell death [4]. The neuropathological diagnosis is divided into six progressive and predictable stages based on the presence of tau [5]. Interestingly, olfactory deficiencies and tau accumulation in the olfactory bulb (OB) have been described in the first stages of disease, even decades before memory impairments appear [6,7].

Somatostatin (SST) is widely expressed in different body tissues, including the brain, where it can be found as a neuropeptide with two different isoforms, SST-14 and SST-28, and it can act through five different SST receptors [8]. SST is mainly expressed in interneurons and has a prominent inhibitory role, controlling the activity of both principal pyramidal neurons and other interneurons, including those expressing parvalbumin and even other SST-expressing interneurons [9,10]. Therefore, SST impairment causes excitatory-inhibitory imbalance, which is involved in diverse neurodegenerative disorders, including AD [11]. During the 1980s, some studies indicated that SST involvement was critical [12]. Moreover, at the beginning of the century, SST was proposed as a key factor in the etiopathogenesis of AD [13]. Recently, SST has been revealed to be the main molecule that binds with Aβ and has been proposed as an effective nucleator during the aggregation of Aβ peptides [14]. However, in recent decades, there has been a lack of research on SST in the human brain [15].

In contrast, glia have gained interest due to their leading role in both neuroprotection and neurodegeneration processes [16,17]. On the one hand, microglial cells are the main cells that eliminate Aβ aggregates [18]. Moreover, astrocytes can prevent Aβ and tau toxicity through the liberation of chaperones [19,20]. In fact, chaperones have been proposed as pharmacological therapies against AD [21]. Nonetheless, glia, pathology, and SST expression in the limbic system in AD remain poorly understood.

## 2. Results

All approaches were performed on human brain samples provided for the Spanish national brain bank network (for details, please see the Materials and Methods section).

### 2.1. Somatostatin Expression in the Olfactory Bulb

**Figure 1:**
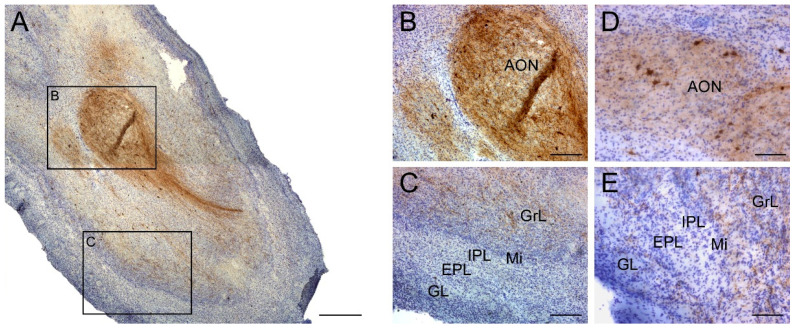
Immunohistochemistry staining for somatostatin in the olfactory bulb. Somatostatin is present in all regions within the olfactory bulb (**A**) with a striking presence in the anterior olfactory nucleus (**B**), and expression was low in the GrL and within the Mi, IPL, EPL and GL external layers in non-AD cases (**C**). Similarly, SST-cells did not show neurodegeneration features in AD cases and were mainly placed in the anterior olfactory nucleus (**D**). In addition, lower intensity of labeling in SST -expressing fibers was observable in the rest of olfactory layers in AD cases (**E**). Note that the number of somatostatin-expressing cells was very low compared with the number of somatostatin fibers. GrL; granule cell layer, Mi; mitral cell layer, IPL; internal plexiform layer, EPL; external plexiform layer, GL; glomerular layer. Scale bar **A**, 500 µm; **B**,**C**, 200 µm.

SST-expressing cells and fibers had a specific distribution within the OB. Most cells and fibers were localized to the anterior olfactory nucleus (AON) or surrounded it (Figure 1A,B). SST expression was also primarily located in the granule cell layer and occasionally reached the glomerular layer (Figure 1C).

**Figure 2:**
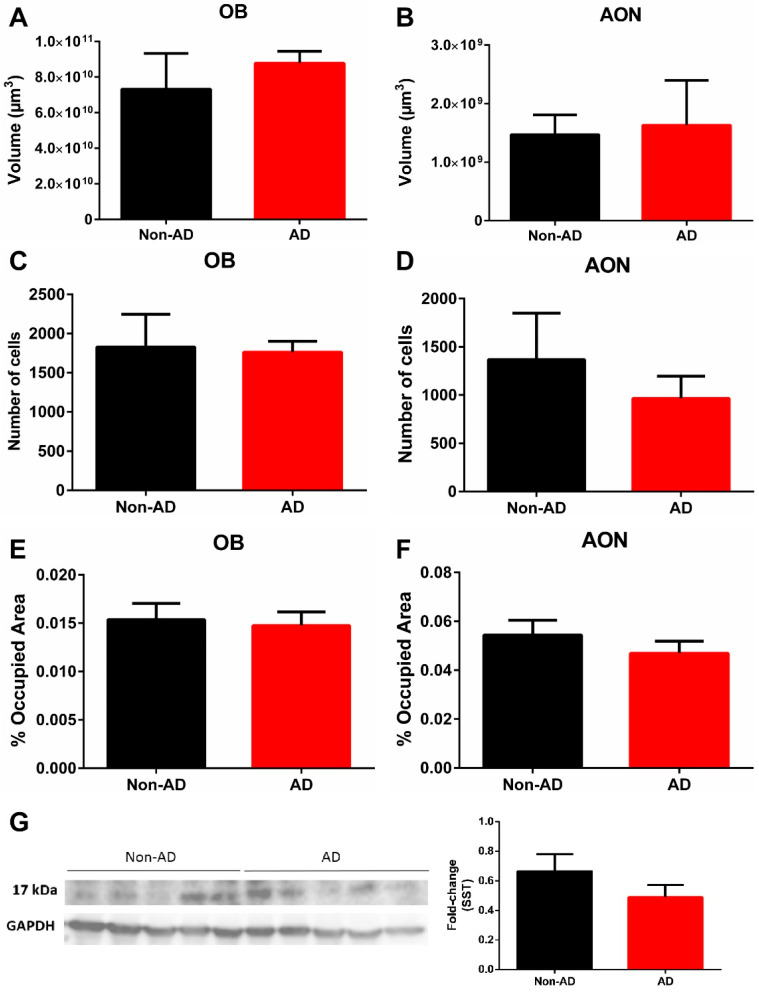
Quantification of volume and somatostatin expression in the olfactory bulb. Neither the olfactory bulb nor the anterior olfactory nucleus showed variation in volume (**A**,**B**). The results also indicated no difference in either the number of somatostatin-expressing cells (**C**,**D**) or the area fraction occupied by somatostatin fibers (**E**,**F**). In addition, Western blot quantifications confirmed no change in somatostatin expression in the olfactory bulb (**G**). For volume, number of cells and somatostatin fibers quantification N = 10 formalin-fixed cases (AD = 5 and non-AD = 5) and 4 sections per case were analyzed. Western blot quantification was performed using N = 20 fresh-frozen cases, AD = 10 and non-AD = 10. See Table 1 and supplementary stereological data for further information.

The results did not show variation in volume in the OB or AON when comparing non-AD and AD cases (Figure 2A,B). In addition, the number of SST-expressing cells did not vary in the OB or AON when comparing non-AD and AD cases (Figure 2C,D). Similarly, the area fraction occupied by SST-positive fibers in the OB and AON did not show significant differences between non-AD and AD cases (Figure 2E,F). Western blot quantification also did not reveal differences in SST expression (Figure 2G).

### 2.2. Somatostatin Expression in the Hippocampus

**Figure 3:**
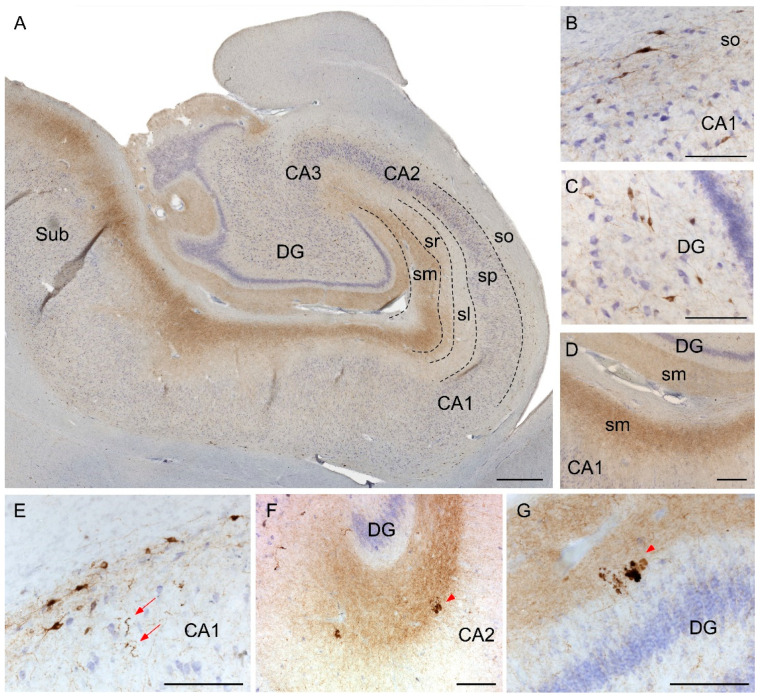
Immunohistochemistry staining of somatostatin in the hippocampus. Mosaic reconstruction of the non-AD human hippocampus showing somatostatin distribution (**A**). In non-AD cases, somatostatin-expressing cells were mainly located in either the stratum oriens in CA regions (**B**) or within the hilus in the DG (**C**) and somatostatin fibers were mainly localized to the molecular layers of CA-DG regions (**D**). The major hallmarks of somatostatin in AD brains were dystrophic neurites (**E**) and aggregates of fibers and cell debris mainly in the molecular layers of CA (**F**) and DG (**G**). CA, cornus amonis; DG, dentate gyrus; Sub, subiculum; so, stratum oriens; sp, stratum piramidale; sl, stratum lacunosum; sr, stratum radiatum; sm, stratum moleculare. Scale bar **A**, 500 µm; **B**–**G**, 160 µm.

The distribution of SST in the Hip differed between its subfields. While cornu ammonis fields CA1-CA3 contained most SST cell bodies in the stratum oriens (Figure 3A,B), SST cells within the dentate gyrus (DG) were randomly distributed (Figure 3A,C). Interestingly, most SST fibers in the Hip localized to the molecular layers of CA1-3 and DG (Figure 3A,D). On the other hand, in AD cases, SST-expressing cells commonly presented dystrophic neurites (Figure 3E). In addition, the other main feature was the presence of cell debris aggregates. Interestingly, the aggregates were most abundant within the molecular layers of CA-DG (Figure 3F,G).

**Figure 4:**
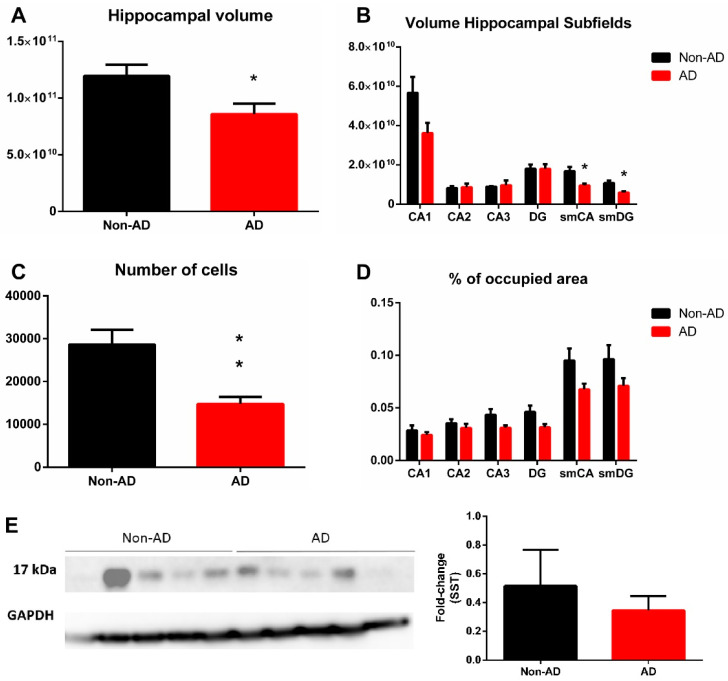
Quantification of volume and somatostatin expression in the hippocampus. Hippocampal volume was reduced in AD (**A**), and the molecular layers of CA and DG regions were specifically involved (**B**). The number of somatostatin-expressing cells was diminished in the whole hippocampus (**C**), but only a tendency (not significant) was observed in the areas occupied by somatostatin fibers (**D**). Western blot quantification did not show a significant reduction in somatostatin expression in the hippocampus (**E**). For volume, number of cells and somatostatin fibers quantification N = 10 formalin-fixed cases (AD = 5 and non-AD = 5) and 4 sections per case were analyzed. Western blot quantification was performed using N = 10 fresh-frozen cases, AD = 5 and non-AD = 5. See Table 1 and supplementary stereological data for further information. * Corresponds to *p*-value < 0.05.

The tissue volumes of non-AD and AD cases were compared at six different regions of interest within the Hip, including CA regions, DG and the molecular layers of the CA regions and DG. The whole Hip underwent a significant volume reduction, *p*-value = 0.0379, t = 2.484 df = 8 (Figure 4A). The specific regions that presented a significant reduction in volume were those where SST occupied most of the area, including the stratum moleculare of the CA, *p*-value = 0.0149, t = 3.088 df = 8 and DG, *p*-value = 0.0097, t = 3.376 df = 8 (Figure 4B). The volumes of the remaining subregions (i.e., CA2, CA3 and DG) did not vary.

In the Hip, the number of SST cells was reduced by 50%, *p*-value = 0.0066, t = 3.640 df = 8 (Figure 4C). Specific analyses of SST fibers indicated that all hippocampal regions presented no significant reduction in the areas occupied by SST (Figure 4D). These results indicate that the number of SST cells is dramatically reduced in the limbic system in AD. However, the reduction in SST fibers was circumscribed to the molecular layers (CA-DG) of the Hip, although without becoming significant. Interestingly, Western blot quantification did not reveal a significant reduction in SST in the Hip (Figure 4E).

### 2.3. Somatostatin, Amyloid-β and Tau

**Figure 5:**
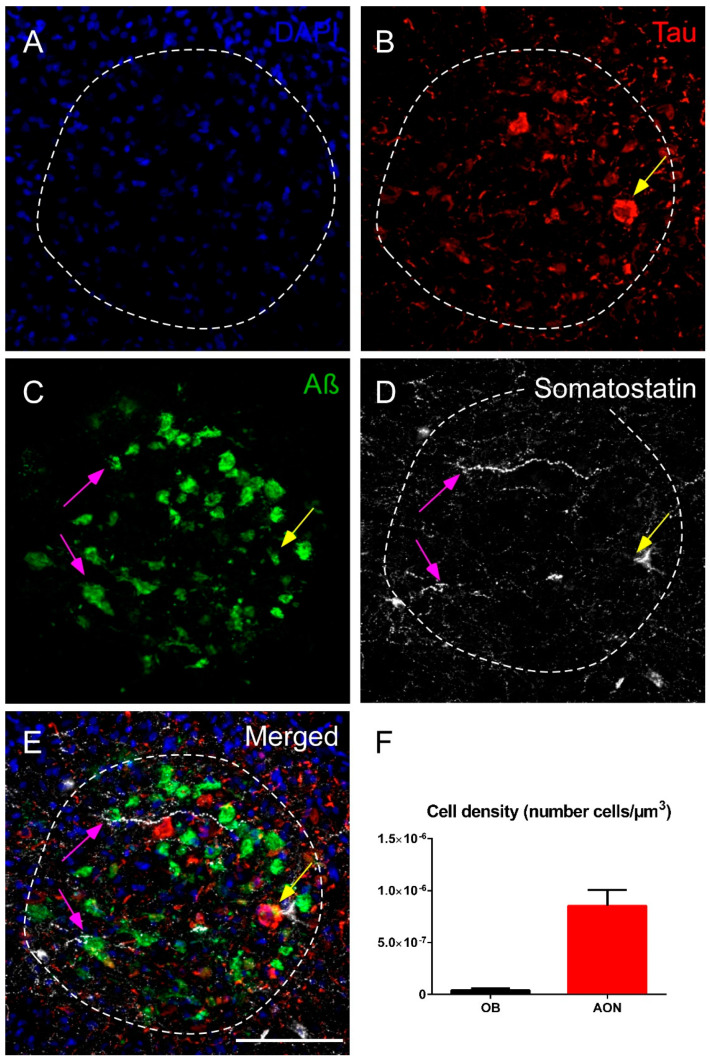
Immunofluorescence staining of somatostatin, tau and amyloid-β in the human olfactory bulb in AD. Representative images show DAPI staining in the anterior olfactory nucleus (**A**), tau (**B**), amyloid-β (**C**) and somatostatin (**D**). Note the elevated expression of amyloid-β and somatostatin and the preferential coexpression of both markers (purple arrows) or, less commonly, the coexpression of the three markers (yellow arrow) (**E**). The cell density indicates that the anterior olfactory nucleus is the main area in the olfactory bulb that expresses somatostatin (**F**). sp, stratum piramidale; sm, stratum moleculare. Scale bar **A**–**E** 100 µm.

In the human OB, the AON is the main area expressing SST, Aβ and tau. Although the three markers were coexpressed in the AON, SST preferentially colocalized with Aβ rather than with tau in AD cases studied (Figure 5A–E). Both tau and Aβ were observed throughout the OB, but the most intense expression was in the AON (Supplementary Figure S1). These results indicate that AON is a key olfactory area with pathology and, interestingly, the main olfactory area where SST is located (Figure 5F).

**Figure 6:**
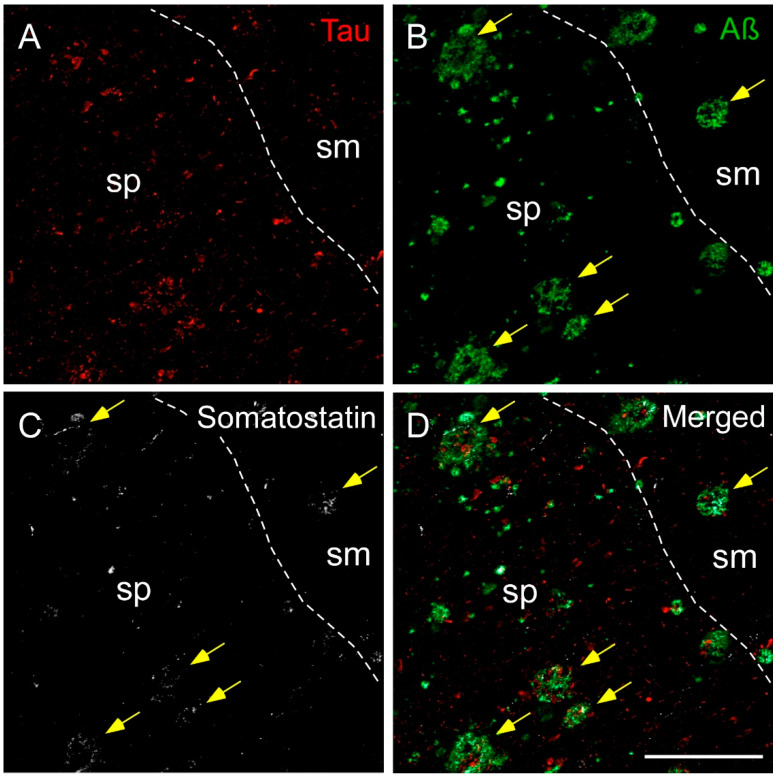
Immunofluorescence staining of somatostatin, tau and amyloid-β in the human hippocampus in AD. Representative images show staining in the CA1 subfield for tau (**A**), amyloid-β (**B**) and somatostatin (**C**). Tau was mainly located in the pyramidal layer (sp), whereas amyloid plaques localized to both the pyramidal layer and the molecular layer (sm). Somatostatin fibers and cell debris were found in both layers, preferentially coexpressing amyloid-β (yellow arrows) (**D**). Scale bar **A**–**D** 200 µm.

In a similar manner to the OB, SST was mainly coexpressed with Aβ in the human Hip samples and rarely with tau in AD cases analyzed (Figure 6). In the Hip, tau labeling was mainly observed in the pyramidal layer (stratum pyramidal) in all subregions, whereas Aβ plaques appeared not only in the pyramidal layer but also in the molecular layer (stratum moleculare) (Figure 6A,B). In addition to tau and Aβ, SST fibers and/or SST cell debris were present in most plaques (Figure 6C,D).

### 2.4. Somatostatin, Amyloid-β and Astrocytes Distribution

**Figure 7:**
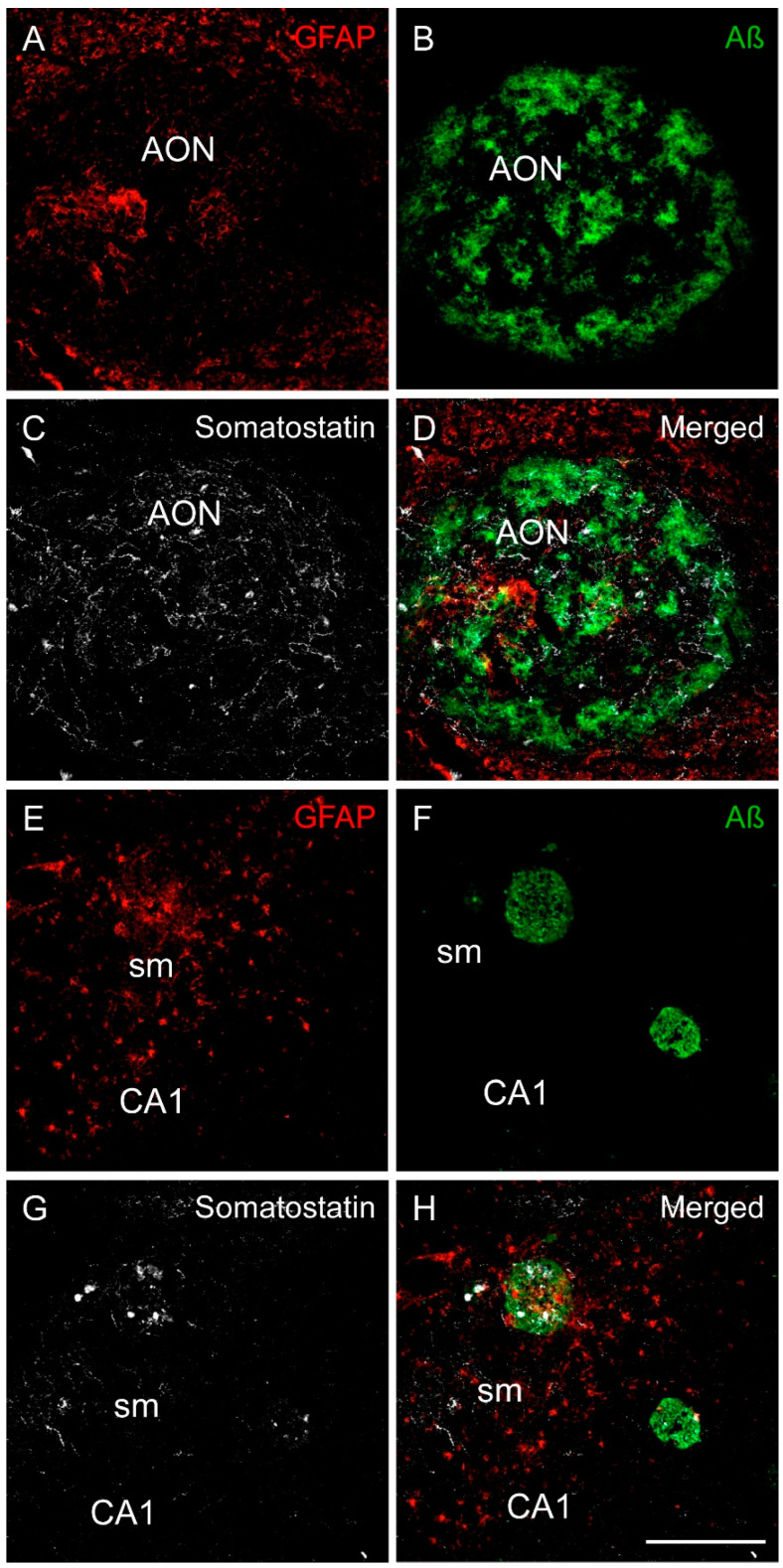
Immunofluorescence staining of somatostatin, amyloid-β and GFAP in the human olfactory limbic systems in AD. Representative images show staining for GFAP (astrocytes in red), amyloid-β (green) and somatostatin (white) in the anterior olfactory nucleus (**A**–**D**) and in the CA1 region of the hippocampus (**E**–**H**). In the anterior olfactory nucleus, there were less astrocytes than in the surrounding area (**A**), whereas amyloid-β (**B**) and somatostatin (**C**) were tightly coexpressed (**D**). In the hippocampus, astrocytes were abundant in the molecular layer of CA1 (**E**). In addition, astrocytes, amyloid-β plaques (**F**) and somatostatin (**G**) were commonly coexpressed in this area (**H**). Scale bar **A**–**H** 200 µm.

GFAP expression differed in the OB and Hip in human brain AD samples. Astrocytes were homogenously distributed across the OB. Surprisingly, although most Aβ accumulated in the AON, only a few astrocytes were observed that formed discrete clusters within the AON compared with the rest of the OB (Figure 7A–D). Therefore, SST and Aβ were abundantly expressed in the AON, but astrocytes were not abundant. On the other hand, astrocytes and SST were localized to the same areas in the Hip, especially in the molecular layers of CA-DG. In addition, the Aβ plaques commonly expressed SST and were surrounded by astrocytes. Interestingly, the Aβ plaques located close to the stratum moleculare coexpressed the highest level of SST and were surrounded by more astrocytes, indicating a specific spatial distribution of these three markers in this hippocampal subregion (Figure 7E–H).

## 3. Discussion

Research on SST in AD peaked during the 1980s and mostly focused on classical histological descriptions. Nonetheless, at that time, SST was highlighted as one of the most affected markers of the disease, particularly in the temporal lobe [22]. However, there was less interest in SST during subsequent decades, and the number of investigations was limited. Here, using unbiased stereology quantification, we discovered differential SST-expression in AD in distinct limbic areas, with molecular layers in the hip being the most affected. We also confirmed that SST is preferentially coexpressed with Aβ rather than with tau. In addition, we discovered a particular spatial relationship between astrocytes, Aβ and SST that varied in the different brain regions studied.

In the OB, most SST-expressing cells and fibers were present in the AON. In addition, tau and Aβ pathologies filled the AON. As a key structure involved in olfactory information processing, the accumulation of the pathology within the AON may underlie olfactory deficits and can correlate with early stages of AD [23]. Although we did not observe an SST reduction in the OB, its coexpression with Aβ was clear. Therefore, SST abnormalities in the olfactory cortex may underlie olfactory impairment [24]. In addition, SST expression in the cortical portion of the AON is drastically reduced [25]. However, in this study, we showed no variation in SST in the bulbar portion of the AON, which may indicate that the pathology progresses in an anterograde manner.

In the Hip, most SST-positive neurons are within the stratum oriens in CA fields, inhibiting pyramidal cells by projecting over distal dendrites located in the molecular layer [26]. Interestingly, in the molecular layer, we found the highest expression of SST, the highest accumulation of astrocytes, and the greatest number of Aβ plaques colocalized with highly expressed SST (Supplementary Figure S2). Even though SST fibers were easy to define, SST labeling in amyloid plaques could correspond to fibers, debris or released SST. Interestingly, no labeling of Aβ nor Tau was found in the stratum oriens, where SST-cell bodies are mainly located. Therefore, the pathology linked to Aβ and Tau is mostly associated with SST fibers or released SST, rather than SST-cell bodies. In addition, the molecular layers of CA1 region also exhibited clusters of microglial cells, which frequently colocalized with Aβ, and SST as well (Supplementary Figure S3). This layer also includes the perforant pathway, which contains axonal projections from the entorhinal cortex to the CA1 subregion, among others, and it is involved in early memory loss [27]. Moreover, these projections may be related to the anterograde spreading of pathological Aβ and tau proteins [28,29]. Recent investigations have confirmed that the dysfunction of SST-expressing interneurons placed in the stratum oriens underlies memory impairment in an AD mouse model [30]. On the other hand, Western blot analyses referred no significant reduction in SST levels in the Hip. Here, we have shown a reduction only in SST cell number but not in SST fibers. In addition, the number of cells is severely reduced as compare with the area occupied by fibers. Hence, we can presume that somatostatin remains in AD brains mainly as fibers. Our results, therefore, suggest that the CA molecular layer is particularly implicated in AD.

**Figure 8:**
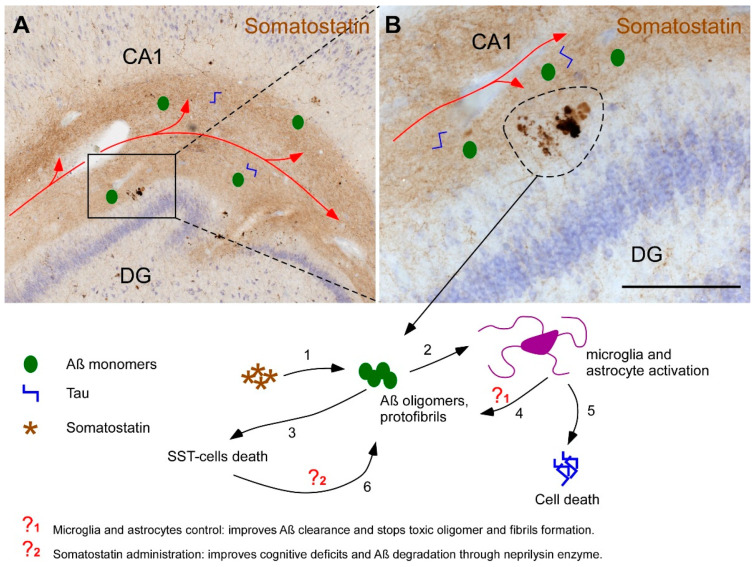
Involvement of somatostatin and glia in the etiology of Alzheimer’s disease. The hippocampus receives afferences from the entorhinal cortex (red line in images **A** and **B**). Pathological proteins Aβ and Tau can spread through this network reaching the molecular layers of CA-DG hippocampal regions. Interestingly, molecular layer contains the highest amounts of somatostatin which in turns favors the oligomerization of Aβ (1). These oligomers and fibrils activate microglial and astroglial populations (2) and cause somatostatin-positive cells death (3). Active astrocytes and microglia have a dual role by promoting Aβ clearance from the extracellular space (4) but also enhancing neuroinflammatory process that breaks cell homeostasis, accumulates intracellular toxic hyperphosphorilated Tau and causes cell death (5). Since somatostatin peptide enriches the expression of the main Aβ degrading enzyme neprilisyn, the death of somatostatin-positive cells, secondarily stimulates the accumulation of Aβ oligomers and fibrils (6). One of the most important therapeutic approaches is, therefore, focused on the regulation of microglia and astrocytes (?_1_), particularly in the management of their state of activation and their capabilities to stop the formation of Aβ oligomers and remove it from the extracellular space as well. Other potential therapeutic strategy is using somatostatin as a transcriptional factor of neprilisyn degrading enzyme (?_2_). Therefore, known somatostatin levels and/or the reactive state of astrocytes could serve for early diagnosis of Alzheimer’s disease. Scale bar **A** = 750 µm, **B** = 200 µm.

Despite controversy regarding the subregions affected early in AD and their utility as biomarkers in clinical diagnosis, CA1 is the region most affected by pathology [31]. The depletion of SST in this and other subregions has been proposed as a key factor in the accumulation of Aβ through downregulation of neprilisyn, which is the main Aβ-degrading enzyme [32]. In fact, transgenic mice intravenously injected with constructs of SST that permeate the blood–brain barrier exhibit increased neprilisyn enzyme levels and decreased Aβ accumulation, suggesting that SST is a useful therapeutic agent [33]. However, astrocytes functionally interact to activate SST cells for the inhibition of pyramidal cells through the overexpression of adenosine A1 receptors [34]. Moreover, astrocytes can slow the accumulation of both Aβ and tau pathologic proteins [20]. In addition, a recent study indicates that blocking microglia stops proinflammatory response, preventing astrocyte reactivity [35]. It is well known that the number of astrocytes does not vary in AD, but they become reactive, inducing neuroinflammatory processes [36]. Our results point to the molecular layers of CA-DG regions, where reactive astrocytes were more abundant (Figure 7E). Thus, this could indicate that the specific subregion might be deeply involved in neurodegenerative processes, such as neuroinflammation, oxidative stress or synaptic dysfunction, all related to reactive astrocytes in AD [37]. However, astrocytes and SST may both have negative implications on disease progression. On the one hand, astrocytes may have neuroprotective functions but, on the other hand, they may also promote neurodegeneration [38]. Moreover, SST can promote the oligomerization of Aβ, which relates it to the etiology of the disease [14] and its administration improves cognitive deficits [39]. Furthermore, SST will be released in the molecular layers of CA-DG at the extracellular space, where it may interact with Aβ isoforms and induce Aβ oligomerization (Supplementary Figure S2). Nonetheless, either for their involvement in neuroprotective actions or for neurodegenerative effects, astrocytes and SST-expressing interneurons might be potential therapeutic targets and/or powerful tools for diagnosis (Figure 8).

In summary, the aim of this work was to stereologically quantify SST cells and fibers in the human limbic system, including the Hip and OB. In addition, using confocal microscopy, we studied the coexpression of SST with Aβ peptide and tau protein, which are the two main pathological features of AD, and the coexpression of SST with astroglia was also examined. The results indicate distinct levels of involvement between the different brain regions studied and specifically reveal the reduction in cell bodies instead of fibers in the hippocampus. In addition, SST was preferentially coexpressed with Aβ but rarely with tau. Interestingly, the SST and astrocyte distributions overlapped. Altogether, we can conclude that SST interneurons are one of the most affected cell types in the Hip in AD. Moreover, SST may act as auspicious sites for Aβ aggregation.

## 4. Materials and Methods

### 4.1. Human Samples

Cases and data were obtained from IDIBAPS, BIOBANK-MUR, BTCIEN and BPA, which were integrated into the Spanish National Biobanks Network. Experimental procedures were approved by the Ethical Committee of Clinical Research at Ciudad Real University Hospital (SAF2016-75768-R and PID2019-108659RB-I00). A total of 50 cases were selected for the study: 30 cases were samples of OB (15 cases of AD and 15 cases classified as non-AD) (Table 1), and 20 cases were samples of Hip (10 cases of AD and 10 cases classified as non-AD) (Table 2). Formalin-fixed samples were used for immunohistochemistry and stereological quantifications, and fresh-frozen samples were used for Western blotting.

To ensure that the experimental conditions were uniform, formalin-fixed samples from different tissue banks were postfixed in fresh phosphate-buffered 4% paraformaldehyde for 45 days. For cryoprotection, blocks were immersed for 48 h in a phosphate buffered (PB) solution of 2% dimethyl sulfoxide (DMSO) and 10% glycerol followed by 48 h in a PB solution of 2% DMSO and 20% glycerol. A freezing sliding microtome was used to obtain 50 μm-thick hippocampal coronal sections and bulbar horizontal sections. Thirteen series from Hip and 5 series from the OB were obtained for each block with 650 μm intervals between sections. The first series was used for Nissl-staining. The remaining series were stored in 24-well plates at −20 °C in 30% ethylene glycol and 20% glycerol in a 0.1 M, pH 7.4 PB solution.

### 4.2. Stereological Quantifications

The OB and hippocampal volumes, the number of SST-expressing cells and the area fraction occupied by SST fibers were quantified under a Zeiss Axio Imager M.2 microscope coupled with stereological software (StereoInvestigator, MBF Bioscience^®^, Williston, VT, USA). Four sections per case were selected for quantification either throughout 2.6 mm of the rostrocaudal axis of the Hip from 16 to 23.9 mm from bregma or for the whole OB [40]. Hippocampal subfields were delimited with a 1× objective (ZEISS Plan-NEOFLUAR 1×/0.025, Ref. 420300-9900) and quantified with a 40× objective (Zeiss Plan-APOCHROMAT 40x/0.95 CorrM27, Ref. 420660-9970).

Volume estimation was carried out using the Cavalieri estimator probe in the case of the OB and with planimetry metrics obtained from the area fraction fractionator (AFF) probe for the different hippocampal regions of interest. The number of SST-expressing cells was quantified using an optical fractionator probe, and SST fiber area fractions were assessed with an AFF probe. For the OB, the dissector height (Z) was established as 14 µm, the guard zones were 2 µm and the counting frame area was 10,000 µm^2^. In the case of Hip, the dissector height was established as 8 µm, the guard zones were 2 µm and the counting frame area was 10,000 µm^2^. For the AFF probe, the counting frame area was 10,000 µm^2^, and 15 µm grid spacing was used for each subregion of both Hip and OB (for details see Supplementary Data 1).

### 4.3. Immunohistochemistry and Immunofluorescence

Tissue epitopes were unmasked by boiling the tissue under pressure for 2 min in citrate buffer. Sections were immersed in formic acid for 3 min and rinsed in phosphate-buffered saline (PBS). Endogenous peroxidase activity was inhibited by incubating in 1% H_2_O_2_ in PBS for 20 min. Sections were preincubated for 1 h with blocking buffer and were incubated overnight at 4 °C with primary antibodies (for details, see Supplementary Table S2). Sections were then incubated in secondary biotinylated anti-rabbit antibody (1:200; Vector Laboratories, Burlingame, CA, USA) for 2 h at room temperature and in avidin–biotin complex (ABC Standard; Vector Laboratories) followed by 0.025% 3.3′-diaminobenzidine and 0.1% H2O2. Sections were mounted, Nissl-counterstained with cresyl violet, dried, dehydrated and coverslipped with DPX (Sigma-Aldrich, Waltham, MA USA).

For immunofluorescence analysis, tissue epitopes were unmasked under pressure for 2 min in citrate buffer and subsequently exposed to UV light for 24 h to reduce autofluorescence. Sections were incubated with blocking buffer for 2 h and primary antibodies (Tau, Aβ, SST and GFAP) overnight at 4 °C. Later, sections were incubated with Alexa 568 anti-rabbit, Alexa 488 anti-mouse or Alexa 647 anti-goat (1:200; Thermo Fisher, Waltham, MA, USA) for 2 h and then with DAPI 0.05% for 10 min at room temperature. Sections were mounted and coverslipped with PVA-DABCO.

### 4.4. Western Blotting

For Western blotting, frozen samples were homogenized with RIPA buffer and micropestles and then incubated for 2 h at 4 °C. Protein extraction was performed by centrifugation at 12,000× *g* and 4 °C for 5 min, and the supernatant was collected. Protein concentrations were quantified using a bicinchoninic acid assay (BCA; Sigma Aldrich). Thirty micrograms of protein for each sample were prepared using 6.25 μL of Laemmli buffer and 0.2 M PBS. Then, samples were loaded on 10% SDS-Tris-Trizma-polyacrylamide gels followed by electrophoresis and transfer to nitrocellulose (OB samples) or PVDF (Hip samples) (Bio-Rad, Hercules, CA, USA) membranes for 30 min at 1.0 A and 25 V constant in transfer buffer (25 mM Tris-HCl, pH 8.8, 192 mM glycine, and 20% methanol). Membranes were stained with Ponceau red, faded with distilled water and blocked in 5% BSA in TTBS (0.1% Tween-20, 0.06 M NaCl, 0.2 M Tris-hydroxymethyl-aminomethane, pH 8.8) for 1 h at room temperature and then incubated with SST primary antibody overnight at 4 °C. After secondary antibody incubation (1:5000 in 1% BSA, Eurogentec, Cultek) for 1 h, the membranes were washed with TTBS and developed with Clarity Western ECL substrate according to the manufacturer’s directions (Thermo Fisher Scientific). GAPDH was used as a housekeeping protein. The images were captured with a Syngene G:Box (GeneSys software) and were then analyzed using ImageJ (Fiji, Gensys software, Cambridge UK).

### 4.5. Statistical Analysis

Statistical analyses were carried out using GraphPad Prism^®^ software (v6.01; La Jolla, CA, USA). Normality and outliers were analyzed by the Kolmogorov–Smirnov test and Rout’s, respectively. When data reached normality, a two-tailed *t*-test was used. In contrast, when data did not reach normality, the Mann–Whitney U test was performed. For multiple comparations, the two-way ANOVA test was used. Level of significance was stablished as α = 0.05. Data are represented as the mean ± standard error of the mean.

## 5. Conclusions

In summary, SST and Aβ have a tight spatial distribution in the limbic system, highlighting the AON in the OB and the stratum moleculare in the Hip, respectively. The molecular layer of CA, in addition, is the most affected region in AD when comparing all analyzed areas. Interestingly, astrocytes and SST are specifically represented in this layer, and Aβ plaques coexpress high amounts of SST and induce the astrocytic response. In conclusion, we define the molecular layer of CA-DG as the main area involved in the accumulation of astrocytes and SST neurodegeneration in AD.

## Figures and Tables

**Table 1:** Human samples of olfactory bulb used in this study. DxAP (anatomopathological diagnostic); PMD (postmortem delay).

Case	DxAP	Sex	Age	PMD (h)	Brain Weight	Braak Stage	Disease Duration (Years)	Cause of Death	Treatment
31	AD	F	90	12	920	VI	-	Acute respiratory failure, respiratory infection and Alzheimer’s disease	Fresh-frozen
32	AD	F	81	6	935	VI	15	Cardiorespiratory arrest	Fresh-frozen
33	AD	F	80	5	1060	VI	8.5	Acute heart failure	Fresh-frozen
34	AD	M	80	21	1300	VI	-	Respiratory failure; bronchoaspiration	Fresh-frozen
35	AD	F	76	11	900	VI	-	Respiratory failure	Fresh-frozen
36	AD	F	83	2	1000	VI	9	Respiratory failure	Formalin-fixed
37	AD	F	85	2	1150	VI	7	Respiratory failure	Formalin-fixed
38	AD	M	75	3	1050	V	4	Multiorgan failure	Formalin-fixed
39	AD	F	84	2	920	V	11	-	Formalin-fixed
40	AD	M	77	6	1060	VI	10	Acute Respiratory infection	Formalin-fixed
41	Non-AD	F	37	9	1200	-	-	Septic Shock	Fresh-frozen
42	Non-AD	F	83	7	1320	-	-	-	Fresh-frozen
43	Non-AD	M	68	4	1350	-	-	Sepsis	Fresh-frozen
44	Non-AD	F	82	4	800	-	-	Cardiorespiratory arrest	Fresh-frozen
45	Non-AD	F	71	7	975	-	-	Respiratory failure	Fresh-frozen
46	Non-AD	F	62	2	1050	-		Multiorgan failure	Formalin-fixed
47	Non-AD	M	58	6	1500	-		Cardiorespiratory arrest	Formalin-fixed
48	Non-AD	M	53	5	1300	-	2	Respiratory failure	Formalin-fixed
49	Non-AD	M	78	4	1100	-	1.5	Respiratory failure	Formalin-fixed
50	Non-AD	M	63	2	1400	-	1.5	Respiratory failure	Formalin-fixed

**Table 2:** Human samples of hippocampus used in this study. DxAP (anatomopathological diagnostic); PMD (postmortem delay).

Case	DxAP	Sex	Age	PMD (h)	Brain Weight	Braak Stage	Disease Duration (Years)	Cause of death	Treatment
1	AD	M	78	5	-	V	-	-	Fresh-frozen
2	AD	M	85	3	1130	VI	4	High digestive hemorrhage	Fresh-frozen
3	AD	M	73	13	1290	V	2	Cardiorespiratory arrest; severe sepsis respiratory focus	Fresh-frozen
4	AD	M	82	4	1105	V	6	Cardiorespiratory arrest; renal insufficiency	Fresh-frozen
5	AD	M	75	6	1080	VI	10	Respiratory infection (Bronchoaspiration)	Fresh-frozen
6	AD	F	91	8	-	V	nd	-	Fresh-frozen
7	AD	F	80	5	1060	VI	8.5	Acute heart failure	Fresh-frozen
8	AD	F	79	15	1195	V	4	Respiratory failure	Fresh-frozen
9	AD	F	81	13	920	VI	20	Multiorgan failure	Fresh-frozen
10	AD	F	81	6	935	VI	15	Cardiorespiratory arrest	Fresh-frozen
11	AD	F	76	11	-	VI	-	-	Formalin-fixed
12	AD	M	69	2	-	VI	-	Multiorgan failure	Formalin-fixed
13	AD	F	67	4	-	VI	-	Pneumonia (Bronchoaspiration)	Formalin-fixed
14	AD	M	67	4	-	VI	-	-	Formalin-fixed
15	AD	F	78	8	970	VI	-	-	Formalin-fixed
16	Non-AD	M	68	4	1350	-	-	Sepsis	Fresh-frozen
17	Non-AD	M	88	0	1285	-	-	-	Fresh-frozen
18	Non-AD	M	40	5	1400	-	-	-	Fresh-frozen
19	Non-AD	M	46	3	610	-	-	Respiratory infection	Fresh-frozen
20	Non-AD	M	43	5	1412	-	-	Septic shock secondary to pneumonia	Fresh-frozen
21	Non-AD	F	49	2	1500	-	-	Cardiorespiratory arrest	Fresh-frozen
22	Non-AD	F	59	4	1500	-	-	Respiratory failure	Fresh-frozen
23	Non-AD	F	57	13	1335	-	-	Respiratory failure	Fresh-frozen
24	Non-AD	F	41	3	1295	-	-	Renal insufficiency	Fresh-frozen
25	Non-AD	F	71	7	975	-	-	Cardiorespiratory arrest	Fresh-frozen
26	Non-AD	M	77	10	-	-	-	-	Formalin-fixed
27	Non-AD	F	82	4	-	-	-	Respiratory failure	Formalin-fixed
28	Non-AD	M	68	4	-	-	-	Cardiorespiratory arrest	Formalin-fixed
29	Non-AD	M	83	13	1630	-	-	Cardiorespiratory arrest	Formalin-fixed
30	Non-AD	M	78	6	-	I	-	-	Formalin-fixed

## Data Availability

All data is included in the main manuscript together with the supplementary data.

## Supplementary Materials

The following are available online at https://www.mdpi.com/article/10.3390/ijms22168434/s1.
