# Supplementary material for: Somatostatin and Astroglial Involvement in the Human Limbic System in Alzheimer’s Disease
Source: Int J Mol Sci. 2021 Aug 5;22(16):8434. doi: 10.3390/ijms22168434 (PMC8395127; doi:10.3390/ijms22168434)
Supplement: Supplementary file 1 [file ijms-22-08434-s001.zip › ijms-1305042-SI.pdf]

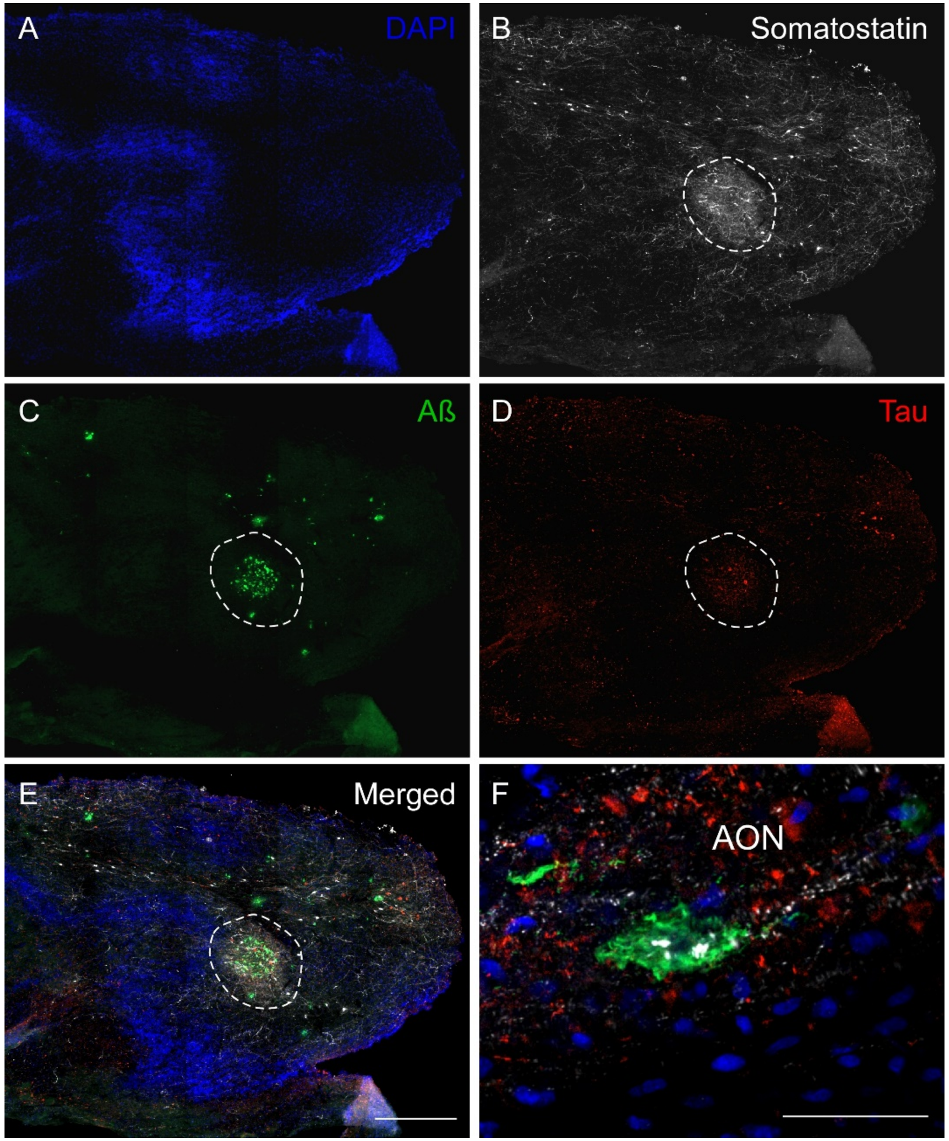

Supplementary Figure S1. Mosaic of the olfactory bulb. Somatostatin can be found in all regions within the olfactory bulb with particular expression in the anterior olfactory nucleus. Note that amyloid-β and tau are localized to different regions across the OB but preferentially coexpress with SST within the AON. Scale bar A-E, 500 µm; F, 50 µm.

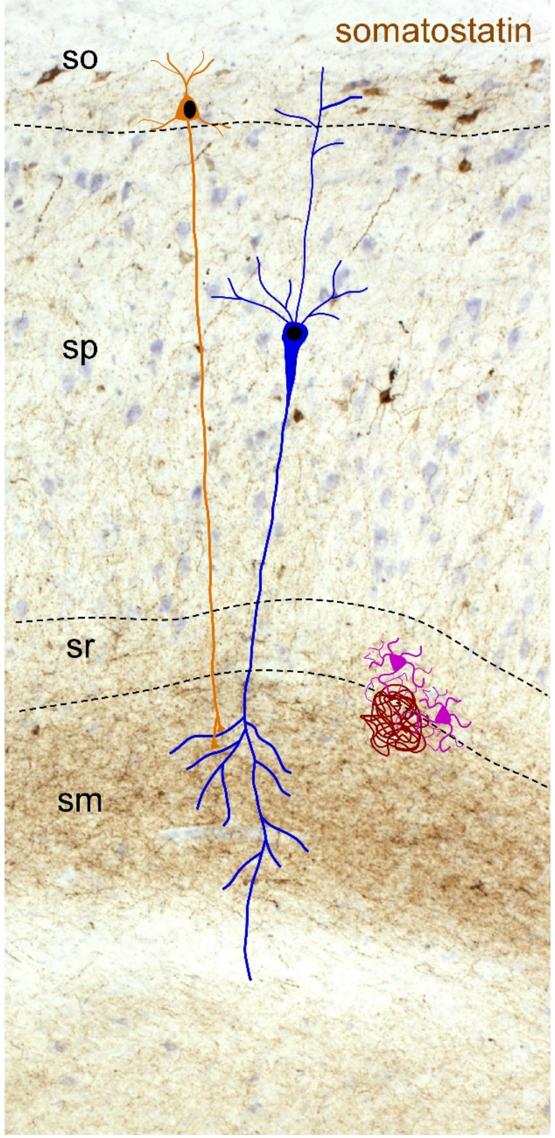

Supplementary Figure S2. Somatostatin distribution in CA1. Representative images show the organization of somatostatin cells within the stratum oriens and their axonal projection over dendrites of pyramidal cells in the stratum moleculare. Most somatostatin fibers are concentrated in the stratum moleculare, which is also where the highest number of astrocytes appear. In addition, amyloid-β plaques in this region are particularly large and present large amounts of SST fibers and SST cell debris (see Figure 6).

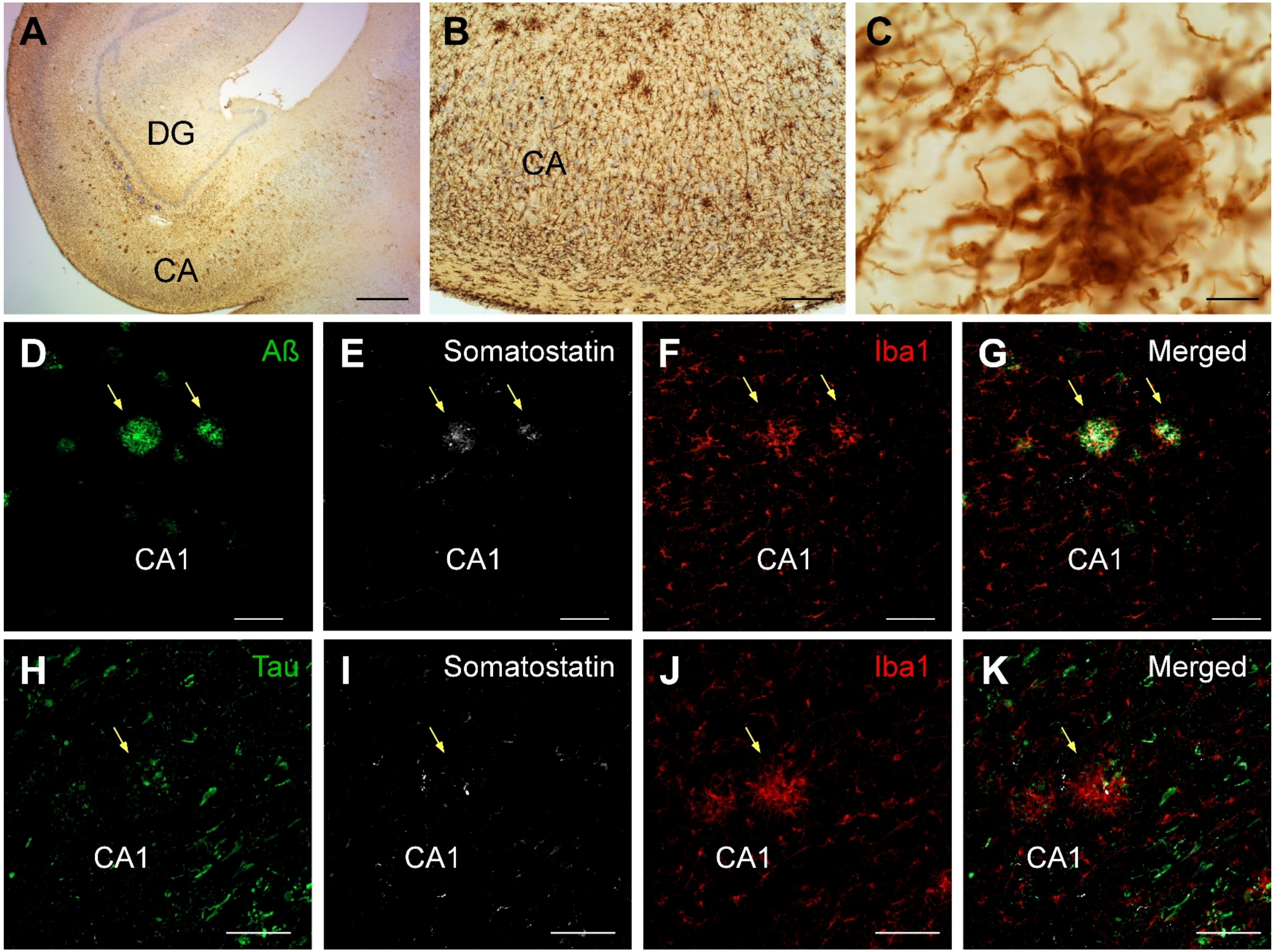

Supplementary Figure S3.

Microglia, somatostatin, and pathology in the hippocampus. Microglia cells labeled with Iba-1 marker are present in the whole hippocampus (A). Interestingly, microglial cells formed aggregates of cells particularly in the molecular layers of CA1 subregion in Alzheimer's brains (B, C). Further, these clusters colocalized in all cases with amyloid-β plaques and somatostatin (D-G) and in less extent with Tau protein (H-K). Scale bars, A = 900 μm; B = 50 μm; C = 10 μm; D-K = 100 μm.

Supplementary table S1. Olfactory Bulb Cavalieri Data

Case	Volume Corrected for OverProjection (mm³)	Coefficient of Error (Gundersen) , m=1	Section Cut Thickness (μm)	Section Evaluation Interval	Grid Size (μm)	Sections	Count
AD cases							
**11**	104,554	0,016	50	13	100	4	16473
**12**	96,0125	0,021	50	13	100	4	15092
**13**	94,5555	0,018	50	13	100	4	14901

**14**	71,0505	0,015	50	13	100	4	11200
**15**	72,77	0,021	50	13	100	4	11482
Non-AD cases							
**25**	12,3225	0,046	50	13	100	3	1872
**26**	54,506	0,017	50	13	100	4	8577
**27**	134,204	0,02	50	13	100	4	21100
**29**	90,108	0,019	50	13	100	4	14171
**30**	74,738	0,03	50	13	100	4	11842

Supplementary table S2. Anterior Olfactory Nucleus Cavalieri Data

Case	Volume Corrected for OverProjection (mm³)	Coefficient of Error (Gundersen), m=1	Section Cut Thickness (µm)	Section Evaluation Interval	Grid Size (µm)	Sections	Count
AD cases							
**11**	2,4075	0,019	50	13	100	4	381
**12**	4,203	0,03	50	13	100	4	673
**13**	1,2115	0,049	50	13	100	4	196

**14**	0,1365	0,08	50	13	100	4	22
**15**	0,204	0,086	50	13	100	4	33
Non-AD cases							
**25**	0,377	0,073	50	13	100	3	57
**26**	1,0305	0,034	50	13	100	4	164
**27**	1,7265	0,036	50	13	100	4	277
**29**	2,201	0,029	50	13	100	4	349
**30**	2,0165	0,045	50	13	100	4	324

Supplementary table S3. Olfactory Bulb Area Fraction Fractionator Data

Case	Area Fraction	Grid Spacing (µm)	Area Associated with grid point (µm³)	Estimated Area (µm²)	Number of Sampling Sites	Sections	Marker Count
AD cases							
**11**	0,0115	15	225	858600	251	4	106
**12**	0,0166	15	225	1336500	243	4	165
**13**	0,0129	15	225	1125900	299	4	139

**14**	0,0193	15	225	1012500	167	4	125
**15**	0,0134	15	225	648000	150	4	80
Non-AD cases							
**25**	0,0278	15	225	486000	79	3	60
**26**	0,0128	15	225	1279800	314	4	158
**27**	0,0125	15	225	842400	215	4	104
**29**	0,0205	15	225	1069200	158	4	132
**30**	0,0127	15	225	963900	221	4	119

Supplementary table S4. Anterior Olfactory Nucleus Area Fraction Fractionator Data

Case	Area Fraction	Grid Spacing (μm)	Area Associated with grid point (μm³)	Estimated Area (μm²)	Number of Sampling Sites	Sections	Marker Count
AD cases							
**11**	0,041	15	225	95175	35	3	47
**12**	0,0498	15	225	206550	65	4	102

**13**	0,0602	15	225	10125	2	4	5
**14**	0,0521	15	225	321975	99	4	159
**15**	0,0313	15	225	50625	23	4	25
Non-AD cases							
**25**	0,0573	15	225	60750	23	4	30
**26**	0,0543	15	225	216675	66	4	107
**27**	0,0518	15	225	220725	59	4	109
**29**	0,0733	15	225	60750	14	4	30
**30**	0,0351	15	225	176175	68	4	87

Supplementary table S5. Hippocampus Ca1 Subregion Area Fraction Fractionator and Volumetric Data

Case	Area Fraction	Grid Spacing (μm)	Area Associated with grid point (μm³)	Estimated Area (μm²)	Number of Sampling Sites	Sections	Marker Count
AD cases							
**36**	0,0146	15	225	558900	113	4	69
**37**	0,0249	15	225	1061100	136	4	131

38	0,0314	15	225	2389500	234	4	295
39	0,023	15	225	1109700	146	4	137
40	0,0271	15	225	1984500	211	4	245
Non-AD cases							
46	0,0179	15	225	60750	240	4	181
47	0,0257	15	225	3499200	405	4	432
48	0,0264	15	225	2016900	252	4	249
49	0,0261	15	225	1822500	231	4	225
50	0,0467	15	225	3353400	221	4	414

Supplementary table S6. Hippocampus Ca2 Subregion Area Fraction Fractionator and Volumetric Data

Case	Area Fraction	Grid Spacing (µm)	Area Associated with grid point (µm³)	Estimated Area (µm²)	Number of Sampling Sites	Sections	Marker Count
AD cases							
36	0,041	15	225	324000	27	4	40

37	0,0219	15	225	267300	41	4	33
38	0,0401	15	225	526500	42	4	65
39	0,0266	15	225	251100	34	4	31
40	0,0244	15	225	583200	73	4	72
Non-AD cases							
46	0,0246	15	225	356400	44	4	49
47	0,031	15	225	510300	50	4	63
48	0,0467	15	225	445500	32	4	55
49	0,0383	15	225	396900	33	4	49
50	0,0367	15	225	461700	41	4	57

Supplementary table S7. Hippocampus Ca3 Subregion Area Fraction Fractionator and Volumetric Data

Case	Area Fraction	Grid Spacing (µm)	Area Associated with grid point (µm³)	Estimated Area (µm²)	Number of Sampling Sites	Sections	Marker Count
AD cases							
36	0,0321	15	225	243000	29	4	30

37	0,0243	15	225	348300	50	4	43
38	0,0294	15	225	388800	44	4	48
39	0,032	15	225	315900	32	4	39
40	0,0378	15	225	1101600	90	4	106
Non-AD cases							
46	0,0257	15	225	356400	43	4	44
47	0,035	15	225	437400	40	4	54
48	0,0514	15	225	704700	44	4	87
49	0,052	15	225	729000	49	4	90
50	0,0529	15	225	785700	49	4	97

Supplementary table S8. Hippocampus DG Subregion Area Fraction Fractionator and Volumetric Data

Case	Area Fraction	Grid Spacing (μm)	Area Associated with grid point (μm³)	Estimated Area (μm²)	Number of Sampling Sites	Sections	Marker Count
AD cases							

36	0,0316	15	225	810000	82	4	100
37	0,0206	15	225	550800	80	4	68
38	0,0377	15	225	1563300	121	4	193
39	0,0299	15	225	591300	61	4	73
40	0,0372	15	225	915300	79	4	113
Non-AD cases							
46	0,0288	15	225	60750	65	4	64
47	0,0348	15	225	1223100	106	4	151
48	0,0581	15	225	1749600	96	4	216
49	0,0521	15	225	1741500	102	4	215
50	0,0572	15	225	1263600	78	4	156

Supplementary table S9. Hippocampus snCA Subregion Area Fraction Fractionator and Volumetric Data

Case	Area Fraction	Grid Spacing (μm)	Area Associated with grid point (μm³)	Estimated Area (μm²)	Number of Sampling Sites	Sections	Marker Count

AD cases							
**36**	0,053	15	225	688500	48	4	85
**37**	0,0609	15	225	1077300	57	4	133
**38**	0,086	15	225	1085400	51	4	134
**39**	0,0705	15	225	810000	45	4	100
**40**	0,0672	15	225	1239300	74	4	153
Non-AD cases							
**46**	0,0657	15	225	1498500	74	4	185
**47**	0,116	15	225	3248100	97	4	401
**48**	0,0751	15	225	2778300	113	4	343
**49**	0,0941	15	225	1571400	55	4	194
**50**	0,1251	15	225	3102300	82	4	383

Supplementary table S10. Hippocampus smDG Subregion Area Fraction Fractionator and Volumetric Data

Case	Area Fraction	Grid Spacing (µm)	Area Associated with grid point (µm^3)	Estimated Area (µm^2)	Number of Sampling Sites	Sections	Marker Count

AD cases							
36	0,0836	15	225	510300	26	4	63
37	0,0647	15	225	704700	48	4	87
38	0,0929	15	225	810000	40	4	100
39	0,0556	15	225	437400	33	4	54
40	0,0581	15	225	680400	46	4	84
Non-AD cases							
46	0,0795	15	225	899100	40	4	111
47	0,0687	15	225	891000	56	4	110
48	0,1022	15	225	2235600	69	4	276
49	0,0865	15	225	1684800	73	4	208
50	0,1454	15	225	2478600	68	4	306

Supplementary table S11. Olfactory Bulb Optical Fractionator Stereological Quantification Data

Case	Total Markers Counted	Number of Sections	Number of Sampling Sites	Coefficient of Error (Gundersen), m=1	Counting Frame Area (XY) (μm²)	Sampling Grid Area (XY) (μm²)	Estimated Population using Mean Section Thickness with Counts
AD cases							
**11**	7	4	888	0,38	10000	99856	1427,17
**12**	7	4	886	0,38	10000	99856	1446,97
**13**	11	4	1082	0,31	10000	99856	1823,38
**14**	11	4	602	0,31	10000	99856	2096,37
**15**	12	4	544	0,31	10000	99856	2015,27
Non-AD cases							
**25**	5	3	291	0,45	10000	99856	1260,07
**26**	16	4	556	0,26	10000	99856	2948,7
**27**	15	4	800	0,26	10000	99856	2547,01
**29**	8	4	1146	0,36	10000	99856	1716,62
**30**	3	4	796	0,58	10000	99856	671,83

Supplementary table S12. Anterior Olfactory Nucleus Optical Fractionator Stereological Quantification Data

Case	Total Markers Counted	Number of Sections	Number of Sampling Sites	Coefficient of Error (Gundersen), m=1	Counting Frame Area (XY) (μm²)	Sampling Grid Area (XY) (μm²)	Estimated Population using Mean Section Thickness with Counts
AD cases							
**11**	22	4	201	0,22	10000	99856	1068,4
**12**	24	4	355	0,21	10000	99856	1375,89
**13**	23	4	131	0,22	10000	99856	1111,91
**14**	-	-	-	-	-	-	-
**15**	7	3	91	0,37	10000	99856	344,37
Non-AD cases							
**25**	3	3	82	0,41	10000	99856	319,23
**26**	-	-	-	-	-	-	-
**27**	20	4	261	0,22	10000	99856	948,39
**29**	34	3	238	0,19	10000	99856	1627,29
**30**	33	3	222	0,18	10000	99856	2573,3

Supplementary table S12. Hippocampus Optical Fractionator Stereological Quantification Data

Case	Total Markers Counted	Number of Sections	Number of Sampling Sites	Coefficient of Error (Gundersen), m=1	Counting Frame Area (XY) (μm²)	Sampling Grid Area (XY) (μm²)	Estimated Population using Mean Section Thickness with Counts
AD cases							
**36**	110	4	1752	0,1	10000	62500	11394,08
**37**	58	4	636	0,13	10000	62500	19540,47
**38**	36	4	867	0,17	10000	62500	11194,3
**39**	55	4	545	0,14	10000	62500	17600,08
**40**	44	4	932	0,16	10000	62500	14038,64
Non-AD cases							
**46**	271	4	4134	0,06	10000	62500	30552,67
**47**	338	4	4096	0,06	10000	62500	40462,54
**48**	211	4	2873	0,07	10000	62500	24779,75
**49**	230	4	3072	0,07	10000	62500	27656,15
**50**	117	4	1979	0,09	10000	62500	19856,44

Supplementary table S13. Primary antibodies detail.

Antigen	Antibody	Manufacturer	Cat. Nº	Dilution	Blocking buffer	Antibody buffer
Immunohistochemistry						
**Iba-1**	Rabbit anti-Iba-1	Wako	019-19741	1:2000	PBS- 0.1% Tx100	PBS- 0.1% Tx100
**GFAP**	Rabbit anti-GFAP	Dako	Z0334	1:10000	PBS- 0.1% Tx100- 10% NHS	PBS- 0.1% Tx100- 10% NHS
**β- amyloid**	Rabbit anti- β- amyloid	Cell Signaling	2454	1:250	PBS- 0.3% Tx100-2%NDS	PBS- 0.3% Tx100-2%NDS
**Tau**	Mouse anti- Tau	Cell Signaling	4019	1:800	PBS- 0.3% Tx100-2%NDS	PBS- 0.3% Tx100-2%NDS
**GFAP**	Goat anti- GFAP	Abcam	ab53554	1:500	PBS- 0.3% Tx100-2%NDS	PBS- 0.3% Tx100-2%NDS
**Somatostatin**	Goat anti-SST	Santa Cruz Biotechnology	sc-7819	1:1000	PBS- 0.3% Tx100-2%NDS	PBS- 0.3% Tx100-2%NDS
Western Blot						
**GAPDH**	Rabbit anti-GAPDH	Cell Signaling	2118	1:1000	5% low-fat milk in TTBS	5% BSA in TTBS
**Somatostatin**	Mouse anti-SST	Santa Cruz Biotechnology	sc-55565	1:500	5% low-fat milk in TTBS	5% BSA in TTBS
